# Clinical Characteristics and Pathogenic Gene Identification in Chinese Patients With Paget’s Disease of Bone

**DOI:** 10.3389/fendo.2022.850462

**Published:** 2022-03-09

**Authors:** Xiaohui Tao, Li Liu, Xingguang Yang, Zhe Wei, Zhongzhong Chen, Ge Zhang, Zhenlin Zhang, Hua Yue

**Affiliations:** ^1^ Shanghai Clinical Research Center of Bone Disease, Department of Osteoporosis and Bone Diseases, Shanghai Jiao Tong University Affiliated Sixth People’s Hospital, Shanghai, China; ^2^ Department of Orthopedic Surgery, Shanghai Jiao Tong University Affiliated Sixth People’s Hospital, Shanghai, China; ^3^ Department of Urology, Shanghai Children’s Hospital, Shanghai Jiao Tong University, Shanghai, China; ^4^ Law Sau Fai Institute for Advancing Translational Medicine in Bone and Joint Diseases, School of Chinese Medicine, Hong Kong Baptist University, Hong Kong, Hong Kong SAR, China

**Keywords:** Paget’s disease of bone, clinical characteristics, *SQSTM1*, whole-exome sequencing (WES), disease-causing gene

## Abstract

**Objective:**

To evaluate the clinical features of sporadic Paget’s disease of bone (PDB) in China and further explore the underlying genetic abnormalities of the disease.

**Methods:**

Clinical characteristics, biochemical indices, bone turnover markers and radiographic examinations of the patients were collected. Genomic DNA was extracted from peripheral blood and whole-exome sequencing was carried out to identify the potential pathogenic genes. The pathogenicity of the variants was thereafter investigated by bioinformatics analysis.

**Results:**

A total of 50 patients (57.20 ± 15.52 years, male/female: 1.63: 1) with PDB were included and the mean onset age was 48.34 years (48.34 ± 17.24 years). 94.0% of the patients exhibited symptomatic patterns described as bone pain (86.0%), elevated skin temperature at the lesion site (26.0%), bone deformity (22.0%) and local swelling (18.0%). The most frequently involved lesion sites were pelvis (52.0%), femur (42.0%), tibia (28.0%), skull (28.0%) and spine (18.0%), respectively. Additionally, 40.0% of them accompanied with osteoarthritis, 14.0% with pathological fractures, and the misdiagnosis rate of PDB was as high as 36.0%. Serum level of alkaline phosphatase was significantly increased, with the mean value of 284.00 U/L (quartiles, 177.00-595.00 U/L). Two heterozygous missense mutations of *SQSTM1* gene (c.1211T>C, M404T) and one novel heterozygous missense mutation in *HNRNPA2B1* gene (c.989C>T, p. P330L) were identified in our study. Moreover, several potential disease-causing genes were detected and markedly enriched in the pathways of neurodegeneration (including *WNT16*, *RYR3* and *RYR1* genes) and amyotrophic lateral sclerosis (ALS, including *NUP205*, *CAPN2*, and *NUP214* genes).

**Conclusion:**

In contrast to Western patients, Chinese patients have an earlier onset age, more severe symptoms, and lower frequency of *SQSTM1* gene mutation (4.0%). Moreover, a novel heterozygous missense mutation in *HNRNPA2B1* gene was identified in one male patient with isolated bone phenotype. As for other genetic factors, it was indicated that *WNT16*, *RYR3*, *RYR1*, *NUP205*, *CAPN2* and *NUP214* genes may be potential pathogenic genes, pathways of neurodegeneration and ALS may play a vital role in the pathogenesis of PDB.

## Introduction

Paget’s disease of bone (PDB) is a focal metabolic bone disease characterized by excessive and aberrant bone remodeling that results in bone pain, bone deformity and pathologic fracture, followed by secondary osteoarthritis and nerve compression syndromes (1, 2). It is well known that there are pronounced geographical and ethnic differences in the prevalence of PDB (3), with the high prevalence in western countries, especially in European descent. Some studies reported intranational regional differences (4–7) and mainly affects people over 55 years (3, 8, 9). However, the disease is extremely rare in Asia and Africa (1, 2, 10–13). For instance, the incidence rate in Japan is merely 0.00028% (2). So far as we know, the literatures on sporadic PDB in Chinese population are confined to case reports (12–15), no systemic study is available with relatively large sample size up to date. In 2012, we reported our study in thirteen sporadic PDB patients (1). However, the study focused mainly on the mutation detection in *SQSTM1*.

It is known that genetic background and environmental factors are involved in the pathogenesis of PDB. Although remains doubtful (16), some environmental determinants have been reported to play a role in the pathophysiology of PDB, such as toxins, animal exposure and measles virus infection (17–20), *etc.* For decades, accumulating evidence have highlighted that genetic factors are important in the pathogenesis of PDB (6, 21–25). Located on human chromosome 5q35, the *sequestosome 1* (*SQSTM1*) gene encodes p62, a scaffold protein with a vital role in both osteoclast differentiation and activity, has been commonly reported among PDB patients (21, 23–25). Additionally, some cases of PDB-like syndromes have been attributed to mutations in *VCP, TNFRSF11A, TNFRSF11B, HNRNPA2B1, HNRNPA1, ZNF687* and *PFN1*, most of which are known to involved in the amyotrophic lateral sclerosis (ALS) and nuclear factor kappa B (NF-kB) signaling pathways (26–32). Recently, several large-scale genome-wide association studies (GWAS) have been conducted to uncover variants at the *CSF1*, *OPTN*, *TNFRSF11A*, *PML*, *RIN3*, *TM7SF4* and *NUP205* loci that may increase susceptibility to PDB (33, 34). For years, we have made great efforts to explore and determine the roles of candidate genes in the development of PDB in Chinese population (26, 27, 31, 35). However, the reported genes could only explain part of the pathogenesis, the pathophysiological mechanism of PDB is needed to be elucidated. Therefore, it is imperative to determine the genetic etiology of PDB and provide guidance for efficient molecular diagnosis. Considering the different genetic background between Chinese and Caucasian population (1, 10–12, 14), the exploration of potential pathogenic genes of PDB in Chinese population also beneficial to explain the pathogenesis of PDB.

In our study, we not only analyzed the clinical characteristics in 50 PDB patients, but also observed the drug efficacy and prognosis by follow-up. Meanwhile, the whole-exome sequencing (WES) was carried out to investigate the underlying gene variants and molecular mechanism of PDB in Chinese population.

## Materials and Methods

### Subjects

The study was approved by the Ethics Committee of Shanghai Jiao Tong University Affiliated Sixth People’s Hospital. All recruited participants provided written informed consent. 50 unrelated Chinese individuals with PDB were enrolled from November 2004 to July 2021, 13 of them participated in our previous study (1). The diagnosis of PDB was established based on standard clinical criteria (36), including clinical characteristics, typical imaging findings and abnormal biochemical/bone turnover markers (BTMs).

First-degree relatives of PDB patients were asked to fill in the questionnaire (medical documentation, whether there are typical symptoms of PDB such as bone pain, bone deformity and local swelling, *etc.)* and samples of blood were collected for biochemical screening. Inclusion criteria for sporadic patients were as follows: No family history of PDB. Meanwhile, there was no abnormal increased serum total alkaline phosphatase (ALP), no PDB symptoms, no abnormal radiograph and radionuclide bone scintigraphy in the other family members.

### Clinical Features and Biochemical Measurements

Clinical data were collected, including sex, onset age, diagnosis age, detailed medical history data (including medication history, previous visiting information and family history). The clinical manifestations (bone pain, bone deformity and local swelling, *etc.)* and related complications (osteoarthritis, pathological fracture, hearing loss, headache, vision impairment, *etc.)* of PDB were recorded. In addition, X-ray images and whole bone scintigraphy with 99^m^Tc-methylene diphosphonate (99^m^Tc-MDP) was performed to determine the severity and the involved bone sites.

Serum ALP, phosphorus, calcium, and other biochemical indices were assessed by a Hitachi 7,600-020 automatic biochemistry analyzer (HITACHI, Japan). Serum intact parathyroid hormone (iPTH), 25-hydroxy-vitamin D (25OHD), β-CrossLaps of type 1 collagen containing cross-linked C-telopeptide (β-CTX) and osteocalcin (OC) were measured by an automated Roche electrochemiluminescence system (Roche Diagnostic GmbH, Germany).

### *SQSTM1* Mutation Screening

Genomic DNA was extracted from 2-mL peripheral blood DNA samples using the Genomic DNA Extraction Kit (Lifefeng Biotech, Shanghai). Primers were designed with Primer3 software (http://bioinfo.ut.ee/primer3-0.4.0/) (37). All eight exons and intron–exon boundaries of the *SQSTM1* gene were amplified by polymerase chain reaction (PCR). Subsequently, the PCR products were purified and sequenced on the ABI3730XL platform with the BigDye Terminator Cycle Sequencing Ready Reaction Kit (version 3.1; Applied Biosystems; Thermo Fisher Scientific, Inc., USA). The sequencing files were analyzed by Polyphred software, and the results were obtained after manual proofreading.

### WES Strategy and Confirmation

Whole-exome capture and high-throughput exon sequencing were performed by BGISEQ-500 sequencing platform on individuals who tested negative for the presence of *SQSTM1* mutations. After filtering, clean reads were aligned to the human genome reference (GRCh37/hg19) with Burrows-Wheeler aligner (BWA) (38, 39). All variants were analyzed and annotated according to the method adopted by Chen (40). We mainly focused on missense [D-mis, predicted to be deleterious by SIFT (41) and PolyPhen2 (42)] and loss-of-function (LoF, including splice acceptor/donor and frameshift indels) variants and further excluded those with a minor allele frequency (MAF) higher than 1% registered in ExAC (http://exac.broadinstitute.org) and the 1000 Genomes Project. In addition, candidate variants were classified according to the guidelines of the American College of medical genetics and genomics and the Association for molecular pathology (ACMG/AMP) (43).

### Statistical Analysis

All statistical analyses were conducted using SPSS 26.0 for Mac (SPSS Inc., USA). The values were expressed as the mean ± standard deviation if data followed a normal distribution; otherwise, they were indicated with median (25th and 75th percentiles). Variables were checked for normality using the Kolmogorov–Smirnov Z statistic. Differences in continuous variables between groups were evaluated using independent-sample *t* tests or Mann-Whitney *U* test. Dichotomous variables were compared using chi-square test or Fisher’s exact test (if n < 5). A two-sided *P* < 0.05 was considered statistically significant.

## Results

### Study Subjects and Clinical Manifestations

All clinical features and laboratory data were summarized in **Tables 1**, **2**. The study cohort consisted of 50 patients (31 males and 19 females) aged 29 to 85 years (age at clinical diagnosis, 57.20 ± 15.52 years). The body mass index (BMI) of all subjects were 23.08 ± 1.78 kg/m^2^. The earliest onset age was 7 years, with a median disease duration of 6.0 years (quartiles, 1.0-12.0 years). There were 68.0% (34/50) of the patients developed symptoms before the age of 55. The ratio of polyostotic lesions to monostotic lesions was 32:18 (1.78:1). No significant differences in clinical and biochemical features between male and female subjects. The distribution of clinical, biochemical and symptomatic characteristics with respect to age and sex-specific sex and age group were summarized in **Tables 3**, **4**.

**Table 1:** Clinical features of sporadic PDB patients in China.

Variables	Value
Age at clinical diagnosis (years) ^a^	57.20 ± 15.52 (range: 29-85)
Age of onset (years) ^a^	48.34 ± 17.24 (range: 7-82)
Disease duration (years) ^b^	6.0 (1.0-12.0)
Gender, male/female (ratio)	31/19 (1.63:1)
Rural districts/urban districts (ratio)	26/24 (1.08:1)
BMI score (kg/m^2^) ^a,c^	23.08 ± 1.78
Symptomatic/Asymptomatic (ratio)	47:3 (15.7:1)
Symptoms/signs at diagnosis (%)	
Bone pain	43 (86.0)
Warmth of skin overlying Pagetic bone	13 (26.0)
Bone deformity	11 (22.0)
Local swelling	9 (18.0)
Complications (%)	
Osteoarthritis	20 (40.0)
Pathological Fractures	7 (14.0)
Hearing loss	3 (6.0)
Headache	2 (4.0)
Visual impairment	2 (4.0)
Bone lesion site	
Pelvis	26 (52.0)
Femur	21 (42.0)
Tibia	14 (28.0)
Skull	14 (28.0)
Spine	9 (18.0)
Scapula	7 (14.0)
Fibula	6 (12.0)
Rib	5 (10.0)
Humerus	3 (6.0)
Radius	2 (4.0)
Clavicle	1 (2.0)

PDB, Paget’s disease of bone; BMI, body mass index.

a Data are showed as mean ± standard deviation.

b Data are showed as median (25th and 75th percentiles).

c Data are not available in three patients.

**Table 2:** Biochemical profile of PDB patients in China.

Biochemical parameters	No.	Gender, (M/F)	Age of onset (years) ^b^	Age at clinical diagnosis (years) ^b^	M/P	Value ^c^
ALP (U/L) ^a^	49	30/19	48.61 ± 17.31 (7-82)	57.63 ± 15.38 (29-85)	17/32	177.00 (287.00–595.00)
β-CTX (ng/L)	35	23/12	44.74 ± 17.86 (7-82)	55.17 ± 15.88 (29-84)	9/26	724.00 (502.00-1170.00)
OC (ng/mL)	35	23/12	44.74 ± 17.86 (7-82)	55.17 ± 15.88 (29-84)	9/26	37.25 (23.40-52.24)
25OHD (ng/mL)	41	30/49	48.61 ± 17.31 (7-82)	57.63 ± 15.38 (29-85)	17/32	22.09 (13.68-26.39)
iPTH, pg/ml	47	29/17	47.24 ± 17.83 (7-82)	56.50 ± 15.22 (29-84)	14/32	36.79 (31.42-55.06)
Calcium, mmol/L	47	29/17	47.24 ± 17.83 (7-82)	56.50 ± 15.22 (29-84)	14/32	2.35 (2.25-2.44)
Phosphorus, mmol/L	47	29/17	47.24 ± 17.83 (7-82)	56.50 ± 15.22 (29-84)	14/32	1.12 (0.94-1.21)

a Except for one patient who was previously treated with zoledronic acid before coming to our unit).

b Data are showed as mean ± standard deviation (range).

c Data are showed as median (25th and 75th percentiles).

PDB, Paget’s disease of bone; No., Number, M/F, male/female; M/P, monostotic/polyostotic; ALP, total alkaline phosphatase; β-CTX, β-CrossLaps of type 1 collagen containing cross-linked; 25OHD, 25-hydroxyvitamin D; OC, osteocalcin; iPTH, intact parathyroid hormone.

Reference range: ALP: 15-112 U/L; β-CTX: 278–540 ng/L (44); OC: 13.07–27.68 ng/mL (44); 25OHD: >30 ng/mL; iPTH: 15–65 pg/mL; Calcium: 2.08–2.60 mmol/L; Phosphorus: 0.80–1.60 mmol/L.

**Table 3:** Sex-specific clinical features and biochemical profile of PDB in China.

	Male	Female	*P*
No.	31	19	-
Age at onset ^a^	45.60 ± 9.00	52.79 ± 17.61	0.212
Age at clinical diagnosis ^a^	56.83 ± 15.84	57.58 ± 15.64	0.839
ALP (U/L) ^a^	466.95 ± 429.59 ^c^	421.95 ± 490.60	0.371
Number of affected skeletal sites ^b^	2 (1.00-4.00)	2 (1.00-3.00)	0.137
Asymptomatic (%)	2 (6.45)	1 (3.23)	0.609
Monostotic (%)	9 (29.03)	9 (47.39)	0.190

No.: Number; ALP, total alkaline phosphatase.

a Data are showed as mean ± standard deviation (range).

b Data are showed as median (25th and 75th percentiles).

c Except for one patient who was previously treated with zoledronic acid before coming to our unit).

^*^p < 0.05 between male and female group.

**Table 4:** Age-specific clinical features and biochemical profile of PDB in China.

Age range (year) ^a^	No.	Age at onset (year) ^b^	Gender ratio (M/F)	Symptomatic/asymptomatic	Monostotic/polyostotic	Number of Affected Skeletal Sites ^c^	ALP (U/L) ^b^ (RF: 15-112 U/L)
<50	17	30.18 ± 10.77	12/5	16/1	4/13	3.0 (1.5-8.0)	768.13 ± 669.72 ^d^
50–59	15	48.53 ± 7.19	9/6	14/1	5/10	2.0 (1.0-4.0)	416.53 ± 401.08
60–69	6	59.33 ± 6.31	1/4	5/0	4/1	1.0 (1.0-2.25)	406.17 ± 475.10
≥70	12	67.41 ± 12.10	8/4	11/1	5/7	2.0 (1.0-2.75)	325.00 ± 380.82

a Divided according to the age of diagnosis.

b Data are showed as mean ± standard deviation (range).

c Data are showed as median (25th and 75th percentiles).

d Except for one patient who was previously treated with zoledronic acid before coming to our unit).

No., Number, M/F, male/female; ALP, total alkaline phosphatase; RF, reference range.

**Figure 1:**
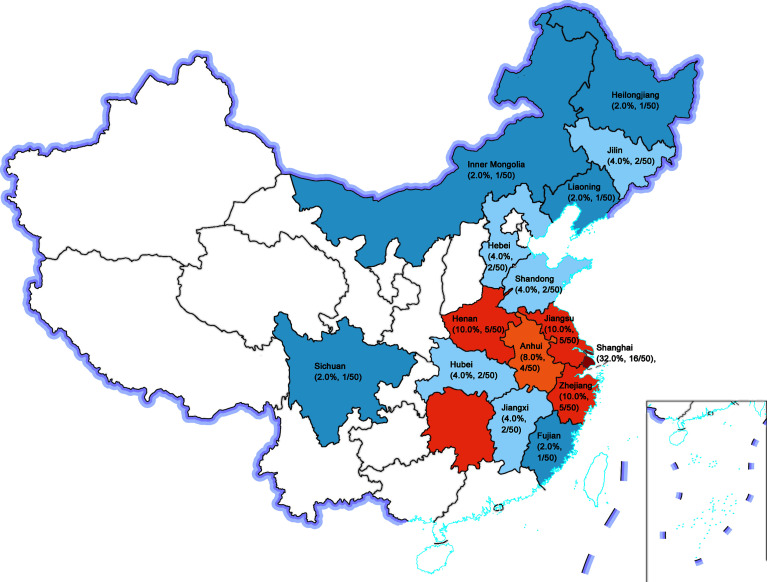
Geographical provenance of the sporadic PDB patients in Chinese population. The figure reported as region (composition ratio, number of patients): Shanghai (32.0%, 16/50), Jiangsu (10.0%, 5/50), Henan (10.0%, 5/50), Zhejiang (10.0%, 5/50), Anhui (8.0%, 4/50), Hubei (4.0%, 2/50), Jilin (4.0%, 2/50), Hebei (4.0%, 2/50), Shandong (4.0%, 2/50), Jiangxi (4.0%, 2/50), Fujian (2.0%, 1/50), Liaoning (2.0%, 1/50), Inner Mongolia (2.0%, 1/50), Sichuan (2.0%, 1/50), and Heilongjiang (2.0%, 1/50).

Geographical provenance of the patients was shown in **Figure 1**. Among them, 48.0% (24/50) were city dwellers and 52.0% (26/50) were rural residents. Moreover, 23 of 50 patients (46.0%) had received the vaccines against measles virus whereas none of them had a history of toxin (such as arsenic and lead, *et al*). A total of 2 patients (4.0%) had contacted with pets (dogs) for at least 1 year before clinical diagnosis.

A total of 94.0% (47/50) of the patients presented with Pagetic symptoms, exhibiting a variety of clinical manifestations with bone pain (86.0%, 43/50), elevated skin temperature overlying Pagetic bone (26.0%, 13/50), bone deformity (22.0%, 11/50), local swelling (18.0%, 9/50), walking difficulty (6.0%, 3/50) and weakness (4.0%, 2/50). Meanwhile, osteoarthritis (40.0%, 20/50) and pathological fractures (14.0%, 7/50) were the most common complications, with 10 fractures occurring in 7 of the 50 PDB patients (54.0%, 7/50), and all the fractures were from the PDB involved sites. Notably, one 32-year-old male patient had 3 fractures (18 years: left femur; 27 years: right fibula; 30 years: left fibula) and one 84-year-old male patient had 2 fractures (57 years: right fibula; 58 years: right tibia) before clinical diagnosis, respectively. The other 5 patients had only one fracture. The predilection sites of pathological fractures were the fibula (4 times), vertebra (3 times), hip (1 time), femur (1 time) and tibia (1 time). No patient required hip arthroplasty for osteoarthritis treatment. Only 6.0% (3/50), 4.0% (2/50) and 4.0% (2/50) of the patients developed hearing loss, headache and vision impairment, respectively. These abnormalities could be attributed exclusively to cranial involvement (45).

Liver and kidney function tests of the 50 patients were in the normal range. Biochemical tests revealed invariably elevated serum ALP levels, with a median value of 284.00 U/L (quartiles, 177.00-595.00 U/L). Serum β-CTX and OC were highly increased, with median values of 868.00 ng/L (563.05-1177.25 ng/L) and 37.65 ng/mL (25.31-56.47 ng/mL), respectively. Furthermore, vitamin D deficiency (defined as 25OHD < 20 ng/mL) was detected in 43.9% (18/41) of the patients, while serum iPTH (92.6%, 25/27), calcium (92.3%, 24/26) and phosphorus (88.5%, 23/26) were within the normal range.

**Figure 2:**
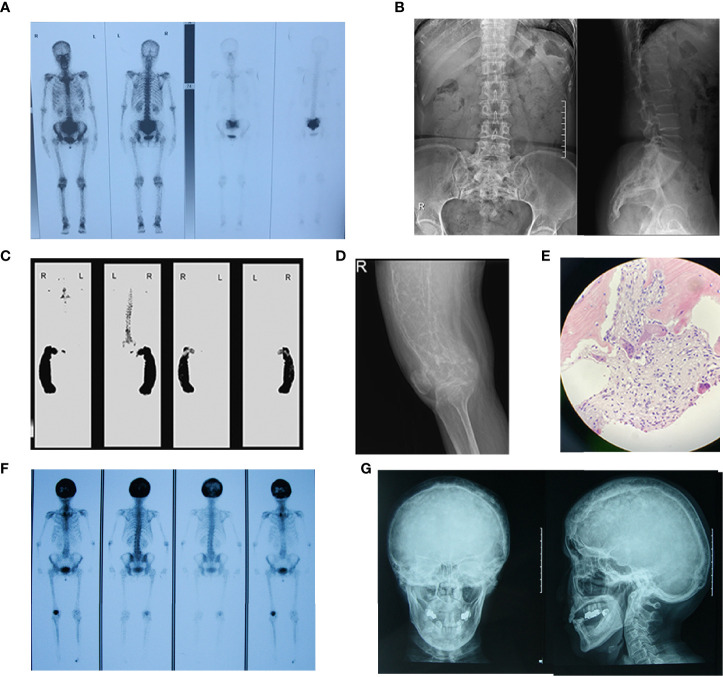
Bone scintigraphy and X-ray images of patients with PDB. **(A)** Bone scintigraphy image depicted with high 99^m^Tc-MDP uptake in sacral vertebrae. **(B)** The frontal and lateral pelvic radiograph showed heterogeneous sclerosis density involving sacral vertebrae. **(C)** The bone scan showed enlargement and bending in a “knife arc” pattern of the left femur, accompanied by high 99^m^Tc-MDP uptake in the left femur. **(D)** The radiograph of the left femur revealed structural changes with significant bowing, cortical destruction and dilation. The trabecular bones were coarse and arrayed irregularly, showing a “towel gourd sac-like” appearance and loss of the normally clear distinction between the cancellous bone and cortical bone. **(E)** Biopsy of the left femoral mass revealed osteoclastic giant cells and bone resorption with fibrous tissue hyperplasia. Patient 12 F Bone scintigraphy image depicted with high 99^m^Tc-MDP uptake in the skull. **(G)** The plain skull film showed craniocerebral thickening with the skull plate barrier disappearing. The cranial suture could not be clearly displayed with the mixture of high-density and low-density areas, and the sclerotic foci showed a dense cotton spherical shadow.

Bone scintigraphy revealed abnormal radioactive concentrations at the involved sites. Patients with polyostotic involvement accounted for 64.0% (32/50). The most common site of involvement was the pelvis (52.0%, 26/50), followed by the femur (42.0%, 21/50), tibia (28.0%, 14/50), skull (28.0%, 14/50) and spine (18.0%, 9/50), scapula (14.0%, 7/50), fibula (12.0%, 6/50), rib (10.0%, 5/50), humerus (6.0%, 3/50), radius (4.0%, 2/50) and clavicle (2.0%, 1/50). X-ray imaging presented a disordered and impaired trabecular bone structure, along with irregular shadows of uneven bone density of the involved bones. In addition to the enlarged and deformed skull, various degrees of thickened cranial diploe and mixed areas of bone destruction and osteosclerosis were observed (**Figure 2**).

**Figure 3:**
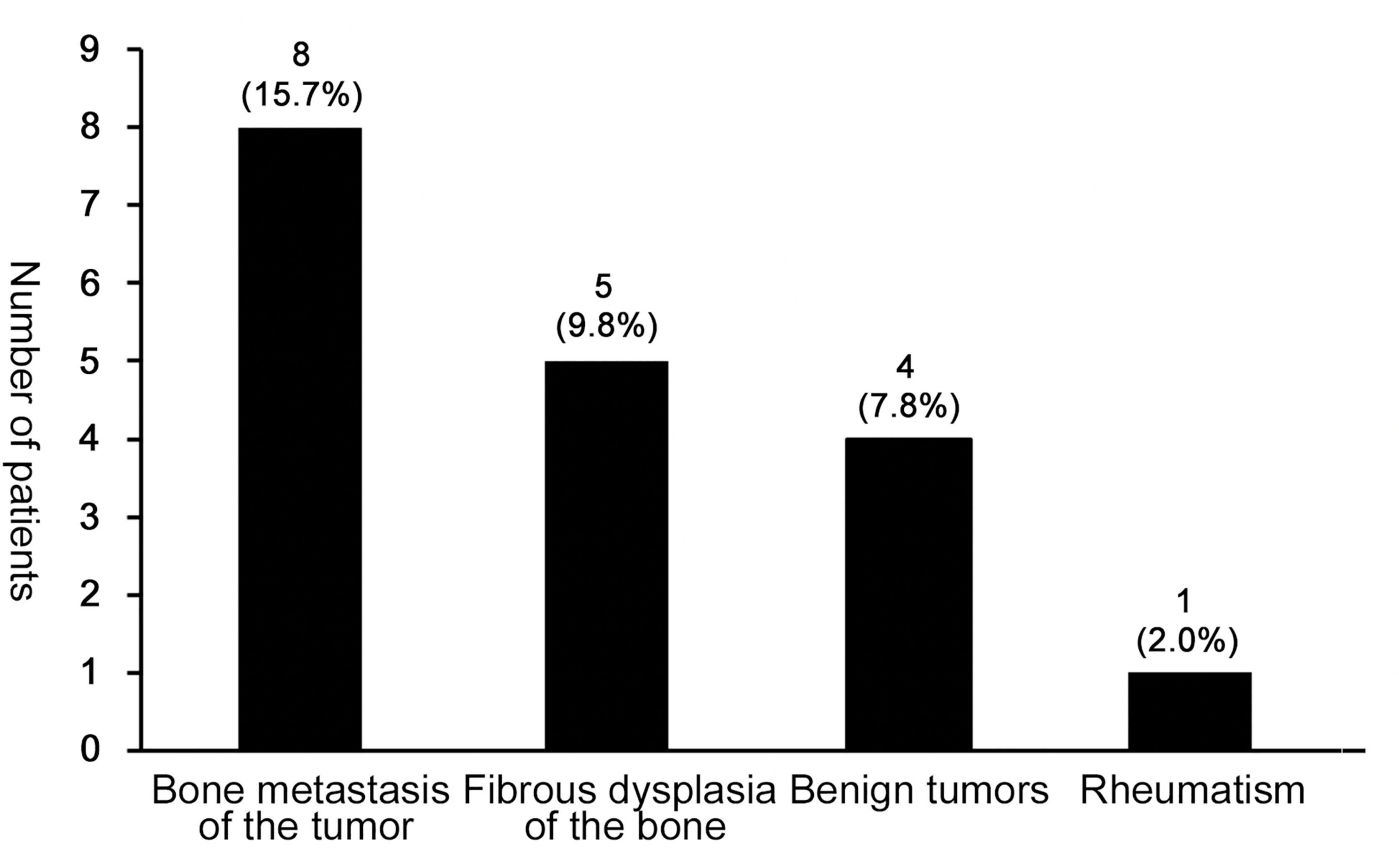
The misdiagnosis condition in patients with PDB. The most frequent misdiagnosed disease was bone metastasis of the tumor (15.7%, 8/50), followed by fibrous dysplasia of the bone (9.8%, 5/50), benign tumors (7.8%, 4/50) and rheumatism (2.0%, 1/50).

Unfortunately, 15 cases (15/50, 30.0%) had received an excisional diagnostic biopsy, whereas 18 patients had once been misdiagnosed and the initial misdiagnosis rate was 36.0%. As was shown in **Figure 3**, patients with PDB were often misdiagnosed as bone metastasis of the tumor, fibrous dysplasia of bone, benign tumor and rheumatism. Among them, one patient was misdiagnosed as benign tumor of the nose and received radiotherapy, which was contraindicated in the treatment of PDB.

### Treatment and Follow-up

41 patients (82.0%) were treated with intravenous zoledronate (5 mg, Aclasta, Novartis Pharma Stein AG, Switzerland or generic drug) yearly. 4 (8.0%) patients with mild symptoms received oral bisphosphonates (70 mg, Alendronate, Merck Sharp & Dohme Ltd, USA) every week. 2 (3.9%) patients refused any treatment. The remaining 3 cases were treated with pamidronate, calcitonin and denosumab, respectively.

Follow-up information, including relevant clinical data and laboratory indictors, was obtained by electronic medical records and retrospective chart review. 84.0% (42/50) of the individuals who received bisphosphonates (3 and 39 individuals received intravenous zoledronate and oral bisphosphonates, respectively) were followed up, with a median follow-up duration of 46.8 months (2.0–119.0 months). During the follow-up period, there were 34, 4 and 1 patients received intravenous zoledronate once, twice, three and four times, respectively. No patient experienced fracture during the period of follow-up. A total of 24 patients (all of them received intravenous zoledronate treatment) underwent BTMs re-examination at different time periods. As shown in **Table 5**, after treatment for 3-months, 6-months and 1-year, the levels of BTMs were remarkably decreased (*P*<0.05).

**Table 5:** Changes in BTMs at different time points after treatment.

Treatment time	No.	Gender (M/F)	Age of onset (years) ^a^	Age at treatment (years) ^a^	Serum ALP (U/L)			Serum β-CTX (ng/L)			Serum OC (ng/mL)		
					Baseline ^b^	treatment ^b^	Change (%)	Baseline ^b^	treatment ^b^	Change (%)	Baseline ^b^	Treatment ^b^	Change (%)
3-month treatment ^c^	6	5/1	37.00 ± 18.30 (29-84)	49.33 ± 19.67 (7-54)	484.00 (180.00-1206.00)	147.50 (65.75-437.25) ^*^	-57.4	1734.50 (642.45-2913.25)	688.35 (80.5425-1740) ^*^	-65.1	89.90 (28.64-161.22)	50.80 (19.13-132.13) ^*^	-28.0
6-month treatment ^c^	12	5/7	41.25 ± 18.64 (12-79-)	51.83 ± 11.28 (34-74)	177.50 (127.50-332.25)	69.50 (53.50-124.00) ^*^	-59.7	882.10 (605.25-1221.00)	262.10 (121.25-721.55) ^*^	-59.4	39.76 (21.46-74.42)	17.67 (14.04-36.20) ^*^	-27.1
12-month treatment ^c^	15	9/1	56.40 ± 13.17 (14-82)	45.67 ± 17.94 (29-83)	287.00 (132.0-0779.00	63.00 (49.00-83.00) ^*^	-73.6	612.00 (507.00-948.20)	251.60 (155.70-457.40) ^*^	-55.4	37.25 (20.68-45.2)	24.20 (16.40-40.82) ^*^	-33.4

a Data are showed as mean ± standard deviation (range).

b Data are showed as median (25th and 75th percentiles).

c including one female patient with SQSTM1 mutation.

^*^p<0.05 when compared to baseline.

BTMs, bone turnover marker; No., Number; ALP, total alkaline phosphatase; β-CTX, β-CrossLaps of type 1 collagen containing cross-linked; OC, osteocalcin.

Reference range: ALP: 15-112 U/L; β-CTX: 278–540 ng/L (44); OC: 13.07–27.68 ng/mL (44).

### Sanger Sequencing for *SQSTM1* Mutation and WES

In addition to the 53-year-old male patient who carried heterozygous M404T (c.1211T>C) in the *SQSTM1* gene[1], we further identified another 54-year-old female who harbored the same mutation (**Supplementary Figure 1**). She complained about the severe episode of low back and hip pain for three months. The diagnosis of PDB was established clinically based on the evaluated ALP level (249 U/L) and the increased radionuclide uptake in the sacral vertebra when she initially visited our institution. Besides, her first-degree relatives had no similar symptoms and were absent from the M404T mutation in the *SQSTM1* gene.

For WES, we achieved an average sequencing depth of 170.36× across the exome target regions, rendering high confidence variant calling. By applying the mentioned criteria, a list of rare deleterious variants that were probably implicated in the pathogenesis of PDB is summarized in **Table 6**. Of interest, we identified 13 patients carrying 15 rare heterozygous variants, all enriched in pathways of neurodegeneration - multiple diseases (2 in *WNT16*, 3 in *RYR3*, 2 in *RYR1*) and ALS pathway (1 in *HNRNPA2B1*, 1 in *NUP205*, 3 in *CAPN2*, 3 in *NUP214*) in Kyoto Encyclopedia of Genes and Genomes database. *In silico* methods such as SIFT and PolyPhen-2 software predicted the variants as likely pathogenic, all of which were validated by Sanger sequencing (**Supplementary Figure 2**) for eliminating false positive. Furthermore, all of the variants were classified as pathogenic according to the ACMG/AMP guidelines. **Table 7** lists the clinical features of the patients with the mentioned rare variants.

**Table 6:** 15 rare disruptive variants in 7 recurrently mutated genes.

Sample	Gene	Chr : Pos	DNA change	Protein change	Zygosity state ^a^	PolyPhen-2 ^b^	SIFT ^c^	ACMG/AMP
A4	*HNRNPA2B1*	chr7:26232882, Exon 10	c.953C>T (NM_031243.3)	p.Pro330Leu (NP_112533.1)	HET	D	T	Pathogenic
A26	*NUP205*	chr7:135279308, Exon 13	c.1844G>C (NM_015035.3)	p.Arg615Pro (NP_055950.2)	HET	D	D	Pathogenic
A27	*CAPN2*	chr1:223905506, Exon 2	c.280C>T (NM_001748.5)	p.Arg94Cys (NP_001739.3)	HET	D	D	Pathogenic
A12 ^d^	*CAPN2*	chr1:223905506, Exon 37	c.479C>T (NM_001748.5)	p.Thr160Ile (NP_001739.3)	HET	D	D	Pathogenic
A11	*WNT16*	chr7:120969800, Exon 2	c.275G>T (NM_057168.2)	p.Cys92Phe (NP_476509.1)	HET	D	D	Pathogenic
A28	*NUP214*	chr9:134004853, Exon 4	c.581C>T (NM_005085.4)	p.A194Val (NP_005076.3)	HET	P	D	Pathogenic
A30	*NUP214*	chr9:134026097, Exon 16	c.2222G>T (NM_005085.4)	p.Arg741Leu (NP_005076.3)	HET	D	T	Pathogenic
A8 ^e^	*NUP214*	chr9:134074060, Exon 29	c.5179G>A (NM_005085.4)	p.Gly1727Arg (NP_005076.3)	HET	D	D	Pathogenic
A12 ^d^	*RYR3*	chr15:33961612, Exon 50	c.5677C>T (NM_001036.6)	p.Arg1893Trp (NP_001027.3)	HET	P	D	Pathogenic
A8 ^e^	*RYR3*	chr15:34030748, Exon 104	c.7613C>T (NM_001036.6)	p.Thr2538Met (NP_001027.3)	HET	D	D	Pathogenic
A29	*RYR3*	chr15:34157399, Exon 37	c.14585G>A (NM_001036.6)	p.Arg4862His (NP_001027.3)	HET	D	D	Pathogenic
A14	*RYR1*	chr19:38993321, Exon 48	c.7789A>G (NM_000540.3)	p.Lys2597Glu (NP_000531.2)	HET	D	D	Pathogenic
A13	*RYR1*	chr19:39006731, Exon 65	c.9559C>T (NM_000540.3)	p.Arg3187Trp (NP_000531.2)	HET	D	D	Pathogenic
A24	*CAPN2*	chr1:223959628, Intron 19	c.2020+1delG (NM_001748.5)	p.X674_splice (NP_001739.3)	HET	NA	NA	Pathogenic
A9	*WNT16*	chr7:120969635, Exon 2	c.81delC (NM_057168.2)	p.S38Pfs*15 (NP_476509.1)	HET	NA	NA	Pathogenic

a Zygosity stats: HET: heterozygous; HO: Homozygous.

b PolyPhen-2 predictions: B, benign; D, probably damaging; P, possibly damaging.

c SIFT predictions: D, deleterious; T, tolerated.

d,e One individual harboring two variants.

NA, not available; ACMG/AMP, the guidelines of the American College of Medical Genetics and Genomics and the Association for Molecular Pathology.

**Table 7:** Clinical features of the 13 affected individuals with 15 rare variants.

Sample	Gene (Protein change)	Age (years)	Sex	Course of disease (years)	Affected skeleton site(s)	Symptoms and signs	ALP (U/L) ^a^
A4	*HNRNPA2B1* (p.Pro330Leu)	32	M	0	skull/spine/pelvis/ribs/bilateral tibia/femur/fibula	bone pain	2349
A26	*NUP205* (p.Arg615Pro)	80	F	20	skull/spine	bone pain/hearing loss	333
A27	*CAPN2* (p.Arg94Cys)	82	M	20	pelvis	null	293
A12	*CAPN2* (p.Thr160Ile), *RYR3* (p.Arg1893Trp)	83	F	1	skull	bone pain	321
A11	*WNT16* (p.Cys92Phe)	42	F	6	skull	bone deformity/swollen	2031
A28	*NUP214* (p.A194Val)	29	M	6	right femur/pelvis	bone pain/bone deformity	445
A30	*NUP214* (p.Arg741Leu)	55	M	26	left femur/bilateral tibia/bilateral fibula	bone pain/bone deformity/weakness	1120
A8	*NUP214* (p.Gly1727Arg), *RYR3* (p.Thr2538Met)	67	M	17	pelvis/right fibula	bone pain	135
A29	*RYR3* (p.Arg4862His)	36	M	8	bilateral femur/left tibia	bone pain/skin temperature increase/swollen/bone deformity	360
A14	*RYR1* (p.Lys2597Glu)	64	F	2	pelvis	bone pain	132
A13	*RYR1* (p.Arg3187Trp)	65	F	0	right femur	bone pain	245
A24	*CAPN2* (p.X674_splice)	34	F	6	skull	bone pain	192
A9	*WNT16* (p.S38Pfs*15)	29	F	1	bilateral tibia/skull/spine/pelvis/bilateral femur/radius/ulna/ribs	bone pain/swollen	785

a Reference range: 15-112 U/L.

Null: asymptomatic.

ALP, alkaline phosphatase, M, male, F, female.

## Discussion

The prevalence of PDB is as high as 5% among people above 55 in Western countries (3, 8, 9); however, it is extremely rare in Asians, with only a few cases reported in China (1, 12–15). To gain better insight into the clinical characteristics and reduce the rate of clinical misdiagnosis among Chinese patients, we analyzed the clinical features in 50 Chinese sporadic PDB patients. Moreover, a comprehensive WES analysis was performed to further explore the potential pathogenic genes associated with PDB. To date, this is the largest cohort and most in-depth study in patients with PDB both at the clinical and molecular level in China (1, 26, 27, 31, 46).

As is shown in **Table 8**, the clinical manifestations of the 50 Chinese PDB patients were summarized and compared to those of PDB reported in different countries (2, 6, 16, 47–52). It is well known that the majority of patients with PDB are asymptomatic in Western countries (53), yet in our cohort, most patients had clinical manifestations. Unlike individuals in Western countries, Chinese patients presented a higher proportion of polyostotic involvements, as well as remarkable clinical manifestations. The reason is that PDB is extremely rare in China, and most clinicians are not familiar with it. The asymptomatic or slightly symptomatic patients are often easily ignored and hardly to be recognized, which could account at least in part for the greater prevalence of polyostotic and symptomatic forms of PDB in our country. However, it is noteworthy that headache, hearing loss and vision impairment are uncommon in Chinese population (45). Similarly, the mentioned neurological complications were rarely found in Japan (2). Previous studies reported that congestive heart failure occurred in 3% of the PDB patients in the USA (54). However, no such cases were observed in our study. Additionally, it was observed that the incidence of fractures in China was roughly the same as that in other countries. While fibula was the most common site of fractures instead of the femur and vertebra (2, 48).

**Table 8:** Comparison of clinical features between PDB observed in China and that of occurred in Western countries (2, 6, 16, 47–52).

	China	Japan	Europe	USA
Lesion sites	Pelvis (50%), femur (41%), tibia (28%), skull (28%), spine (18%), scapula (14%), fibula (12%), rib (10%), humerus (6%), radius (4%), clavicle (2%)	Pelvis (55%), spine (32%), femur (27%), skull (20%), tibia (15%)	Pelvis (35-68%), spine (34-53%), femur (32-50%), skull (23-38%), tibia (30-34%), humerus (11-16%), ribs (7%), radius (0-3%), scapula (2%)	Pelvis (59-67%), spine (30-41%), femur (22-31%), skull (7%), tibia (5%), humerus (2%), radius (0-1%)
Age at diagnosis (years)	Average 57	Average 65	Average 69-75	Average 70-71
Age of onset (years)	Average 48	NA	Average 57	Average 70
Gender ratio (male to female)	1.63/1	0.86/1	1.03-1.5/1	1.2/1
Monostotic (%)	36%	50%	19-72%	28%
Asymptomatic (%)	6%	25%	82%	42-65%
Symptoms/signs at diagnosis	Bone pain (86%), warmth of local skin (26%), bone deformity (22%), swelling (18%), difficulty walking (6%)	Lumbago (24%), buttock pain (14%), coxalgia (23%), gonalgia (9%), bone deformity (19%)	Bone pain (18-67%), bone deformity (9-62%), fractures (7-14%), swelling (9%)	Bone pain (42%), bone deformity (2-8%), warmth of local skin (2%)
Complications	Osteoarthritis (37%), fractures (14%), hearing loss (6%), headache (4%), vision involvement (4%)	Hearing loss (6%), fractures (10%), Sarcoma (1.8%), hip arthroplasty (0.6%)	Headache (19-37%), hearing loss (34%), vision involvement (43%)	Hearing loss (67%), fractures (3-10%),
Familial case reported	Yes	6.3%	12-26%	NA
Malignant transformation	No	NA	0.7-4.8%	0.4%

NA, Data not available.

Of interest, most Chinese patients were early onset, with 66.7% (34/50) of the patients developed symptoms before the age of 55 years, while PDB developed more common in people above 55 years in Caucasian population (51). This may be related to the different genetic backgrounds and need to be further investigated. In addition, the misdiagnosis rate of the disease was as high as 36.0% in Chinese patients and was often misdiagnosed as bone metastasis of malignant tumors and fibrous dysplasia, *etc.* As a result, 30.0% of the patients underwent bone biopsy, which is unnecessary for diagnosis of PDB in daily clinical practice (36, 45, 55, 56). Likewise, in 2005, Hashimoto *et al.* demonstrated that bone biopsy was conducted for diagnosis in 55% of the cases in Japan (2). This tendency in application of invasive examination methods also partly reflects the fact that clinicians are unfamiliar with the disease due to the rarity of PDB in Asia, and our main consideration in diagnosis was to exclude possible malignant bone tumors. Therefore, a full understanding of the clinical characteristics and pathogenesis of the disease is key to reducing misdiagnosis and providing timely treatment to patients. It cannot be ignored that the prevalence of the disease is likely to be underestimated in China due to the lack of understanding of the disease by clinicians. In addition, our long-term follow-up results showed that PDB patients responded well to bisphosphonates. Most patients only required zoledronic acid once a lifetime to avoid clinical relapse [defined by recurrence of bone pain (36)]. Therefore, if the patients were correctly diagnosed, the disease would be quickly and effectively controlled, and the symptoms of patients would be greatly alleviated.

Compared with the high proportion (15–26%) of familial aggregation in Western countries (48), sporadic patients are more common in China. Mutations in *SQSMT1* had been identified in 40-50% of familial cases and in 9-20% of sporadic individuals in Western countries (23, 24, 57). However, in our study, only 4.0% of the patients carried *SQSMT1* mutations, which was much lower than that of in Europe (23–25). Both patients harbored the relatively rare heterozygous identical mutation (M404T) in *SQSTM1* instead of the most prevalent P392L PDB-causing mutation in the Caucasian population (21, 23, 24, 58). Taken together, the above data indicated that different genetic backgrounds did exist across various ethnicities, and the M404T mutation in *SQSTM1* could be a mutational hotspot in Chinese patients. Whether it represented a founder effect in the Chinese Han nationality needs haplotyping analysis to explore and confirm (6).

**Figure 4:**
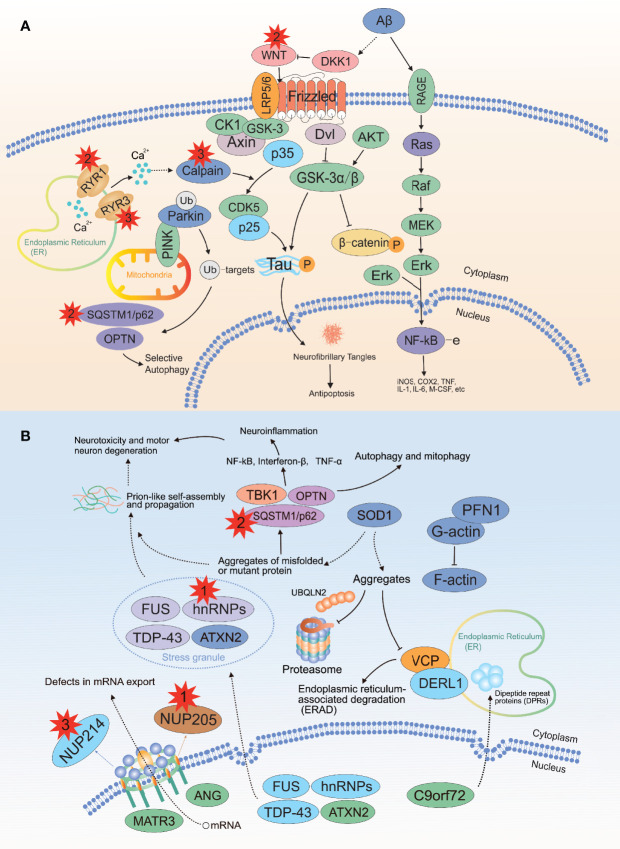
Recurrent damaging variants of the neurodegeneration and ALS pathways in PDB. **(A)** There were two, three, and two mutations located in the *WNT16*, *RYR3* and *RYR1* genes, respectively. **(B)** There were one, one, three and three mutations located in the *HNRNPA2B1*, *NUP205*, *CAPN2* and *NUP214* genes, respectively. ALS, amyotrophic lateral sclerosis; The red explosion patterns and numbers indicate the germline mutation type and frequency, respectively. The figure was produced by Adobe Illustrator Software (Adobe Systems, San Jose, USA).

A well-known feature of PDB is the increased osteoclast activation. Hence, we have focused primarily on the RANKL-induced NF-κB signaling pathway for a long time. In addition to *SQSTM1*, our study identified several rare variants (1 in *HNRNPA2B1*, 1 in *NUP205*, 2 in *WNT16*, 3 in *RYR3*, 2 in *RYR1*, 2 in *CAPN2*, and 3 in *NUP214*) highly related to PDB using in silico tools. Enrichment analysis indicated that the above genes were enriched in neurodegeneration and ALS pathways (**Figure 4**), which were possibly linked to the pathogenesis of PDB. This is of particular interest because PDB, inclusion body myopathy (IBM), together with frontotemporal dementia (FTD)/ALS lead to an inherited pleiotropic group of inherited disorders, coined the term multisystem proteinopathy (MSP), which is characterized by progressive degeneration of brain, muscle and bone (59, 60). It is widely accepted that MSP is attributed to mutations in *VCP*, and the acronym IBMPFD is commonly utilized for the above-mentioned complex phenotypes (59, 60). Notably, the syndromes also show genetic overlap with neurodegeneration, and ALS is classified as a neurodegenerative disease. Along with the rapid development of high-speed sequencing technologies, MSP is genetically linked to mutations in the *PFN1*, *HNRNPA1*, *HNRNPA2B1*, *SQSTM1* and *MATR3* genes (59). Coincidentally, MSP shared genetic overlap with isolated PDB, ALS and other neurodegenerative diseases. In the above diseases, aging is a risk factor (29, 36, 55, 59), no exception for PDB. Till now the relationship between the above genes and diseases remains unclear, further studies should be performed to elucidate the underlying mechanisms related to MSP and isolated PDB.

**Figure 5:**
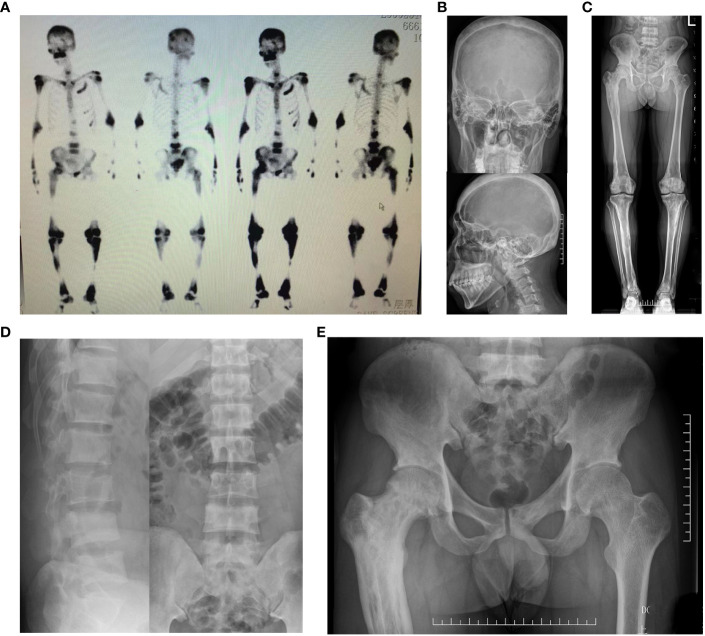
Whole-body bone scintigraphy and radiographic findings of the patient with *HNRNPA2B1* mutation. **(A)** Whole-body bone scintigraphy of the patient showed radionuclide uptake in multiple bones. **(B)** The X-ray of skull showed the thickened skull plate barrier, and the slight enlarged skull bone with diffuse sclerotic foci. **(C)** The radiograph of the bilateral lower extremities revealed the heterogeneous density in both lower limbs with patchy areas of osteosclerotic, and irregular destruction of the cortical bone. **(D)** The lumbar spine radiographs showed the increased density in vertebral bodies with slight vertebral compression. **(E)** The pelvic radiograph showed the increased sclerosis sites in pelvis.

*HNRNPA2B1* encodes a ubiquitously expressed RNA-binding ribonucleoprotein that acts as a binding partner of TDP-43. Accumulating evidence has highlighted those mutations in the *HNRNPA2B1* gene are highly associated with MSP, of which PDB is a component (29, 59). In 2017, the heterozygous missense mutation (c.929C>T, p. P310L) in *HNRNPA2B1* gene was identified as causative of isolated PDB in a large Chinese pedigree by Xia *et al.* Consistent with this, we identified another novel heterozygous mutation in the *HNRNPA2B1* gene (c.989C>T, p. P330L) in one 32-year-old male with sporadic PDB with a pure bone phenotype. He presented with a severe episode of low back and hip pain for 2 months. The bone scintigraphy indicated increased radionuclide uptake in multiple bone lesions, and X-ray examination showed sclerosis at different levels and ranges in the affected bones, accompanied with an uneven bone density (**Figure 5**). It is of interest to note that all cases, no matter in Xia’s or our study, presented with relatively early onset (17, 31, 34, 32 years), significantly elevated ALP (1592, 6724, 2042, 2349 U/L) and recurrent fractures (4, 4, 0, 3 times). Taken together, these data indicated that *HNRNPA2B1*-mutated patients showed a severe symptom, with a remarkable numbers of affected bone sites and increased risks of fractures. It was suggested that PDB patients with severe phenotypes and repeated features, even absence of IBM, FTD, and ALS, should be paid attention to the *HNRNPA2B1* gene in molecular diagnosis.

The nuclear pore complex (NPC) is composed of approximately 30 diverse proteins coined nucleoporins (Nups), including nucleoporin 205 and 214, which are encoded by the *NUP205* and *NUP214* genes, respectively (61). Although the role of the above two genes in bone remains unclear, a large-scale GWAS confirmed the strong signal association of rs4294134 within *NUP205* with PDB (33). Of interest, we identified a missense mutation p.Arg615Pro (c.1844G>C) in a 80-year-old woman, who had exhibited relatively severe PDB symptoms with headache and hearing loss for 20 years. GWAS performed by Albagha *et al.* also demonstrated that the 7q33 locus is susceptible to PDB (33); consistently, our study detected *WNT16*, which locates within the 7q31 locus and encodes the secreted cysteine-rich glycoproteins that have been implicated in cortical bone homeostasis (62). *WNT16* has been previously identified by a GWAS to be associated with BMD, cortical bone thickness and fracture susceptibility (63). Coincidentally, the 29-year-old woman carried the deletion variant p.S38Pfs*15 (c.111delC) in *WNT16* suffered from vertebral compression fractures and local cortical destruction both in tibia and fibula, with an extremely high level of ALP (785 U/L). Moreover, the missense variant p.Cys92Phe (c.275G>T) in *WNT16* was also found in one 44-year old woman, she presented with progressive temporal bone protrusion and raised ALP level for six years (from 1457 U/L to 2031 U/L). The X-ray examination showed that skull was flocky in appearance without sites involved. Both variant sites were classified to be likely pathogenic mutations by ACMG/AMP. Taken together, the underlying relationship between *WNT16* and PDB is worth exploring. Moreover, the recurrent mutant genes in our cohort such as *RyR1*, *RyR3* and *CAPN2* are linked to calcium signaling (64, 65). Although the relationship between them and bone metabolism is still unknown, evidence has been reported that Ca^2+^ signaling activates osteoclast precursor differentiation but inhibits resorption in mature osteoclasts (66).

We have considered the possible limitations of our study. As noted above, the sample size of single-center research was still limited due to the rarity of PDB in China, and multiple-center and larger sample size studies are required to provide a more comprehensive characterization of the disease. Second, as a hospital-setting study, the proportion of symptomatic patients may be overestimated compared with the “real-life” characteristics of PDB. In fact, as an authoritative tertiary care level center devoted to bone disease, our findings may underestimate non-skeletal manifestations. Furthermore, it was insufficient to validate the causality between variants and PDB by only computer algorithms and prediction software, and rigorous experimental assays were required before defining a pathogenic variant (32). Also, we may not identify the presence of unknown modifier genes (32). In the future, studies with wider geographic distribution and larger sample capacity are needed to fully clarify our findings, and demonstrate a more comprehensive picture of PDB in Chinese patients.

Overall, due to the rarity of PDB in Asia, our study represents the first comprehensive and largest sample of patients recruited in China to date, both in terms of clinical evaluation and molecular processes. The detection rate of sporadic PDB patients was extremely low, and only 3 of 50 patients carried the known pathogenic gene mutations (including 2 patients with *SQSTM1* mutation and 1 patient with *HNRNPA2B1* mutation) in our cohort. Besides, we detected other 14 potential pathogenic gene variants, which may have an impact on accurate molecular diagnosis, genetic screening and provide further support for researchers to explore PDB pathogenesis.

## Data Availability Statement

The datasets presented in this study can be found in an online repository. The names of the repository and accession number are as follows: National Omics Data Encyclopedia (NODE) [accession: OEP003182]; https://www.biosino.org/node/project/detail/OEP003182.

## Ethics Statement

The studies involving human participants were reviewed and approved by the Ethics Committee of Shanghai Jiao Tong University Affiliated Sixth People’s Hospital. The patients/participants provided their written informed consent to participate in this study. Written informed consent was obtained from the individual(s) for the publication of any potentially identifiable images or data included in this article.

## Author Contributions

ZZ, GZ and HY contributed to conception and design of the study. XT wrote the first draft of the manuscript. LL wrote sections of the manuscript. XT, LL, GY and ZW collected the samples and organized the database. ZC analysed and interpreted the raw data. ZZ and HY contributed to funding acquisition. All authors contributed to the article and approved the submitted version. XT and LL were co-first authors.

## Funding

This work was supported by the National Key R&D Program of China (No. 2018YFA0800801), National Natural Science Foundation of China (NSFC) (No. 81770874 and 81974126); the Clinical Science and Technology Innovation Project of Shanghai Shenkang Hospital Development Center (No. SHDC12018120) and Shanghai Key Clinical Center for Metabolic Disease, Shanghai Health Commission Grant (No. 2017ZZ01013).

## Conflict of Interest

The authors declare that the research was conducted in the absence of any commercial or financial relationships that could be construed as a potential conflict of interest.

## Publisher’s Note

All claims expressed in this article are solely those of the authors and do not necessarily represent those of their affiliated organizations, or those of the publisher, the editors and the reviewers. Any product that may be evaluated in this article, or claim that may be made by its manufacturer, is not guaranteed or endorsed by the publisher.

## Supplementary Material

Supplementary Figure 1 Sequencing analysis of *SQSTM1* in patients and healthy controls. **(A)** A 54-year-old woman harbored a heterozygous c.1211T>C transversion on exon 8 in *SQSTM1*, resulting in p. Met404Thr (NM_003900.5); **(B)** No M404T mutation was detected in healthy controls.

Supplementary Figure 2 Sequencing traces of the variants in the *WNT16*, *RYR3*, *RYR1*, *HNRNPA2B1*, *NUP205*, *CAPN2* and *NUP214* genes. **(A)** Patient 4 (32 years, male) carried a heterozygous c.989c>T substitution in exon 10 of the *HNRNPA2B1* gene, resulting in p. Pro330Leu (NM_057168.2); **(B)** Patient 26 (80 years, female) carried a heterozygous c.1844G>C substitution in exon 13 of the *NUP205* gene, resulting in p. Arg615Pro (NM_015035.3); **(C)** Patient 27 (82 years, male) carried a heterozygous c.280C>T substitution in exon 2 of the *CAPN2* gene, resulting in p. **(D, I)** Patient 12 (83 years, female) carried heterozygous c.479C>T and c.5677C>T substitutions in the *CAPN2* and *RYR3* genes, resulting in p. Thr160Ile (NM_001748.5) and p. Arg1893Trp (NM_001036.6); **(E)** Patient 4 (42 years, female) carried a heterozygous c.275G>T substitution in exon 2 of the *WNT16* gene, resulting in p. Cys92Phe (NM_057168.2); **(F)** Patient 28 (29 years, male) carried a heterozygous c.581C>T substitution in exon 4 of the *NUP214* gene, resulting in p. A194Val (NM_005085.4); **(G)** Patient 30 (55 years, male) carried a heterozygous c.2222G>T substitution in exon 16 of the *NUP214* gene, resulting in p. Arg741Leu (NM_005085.4); **(H, J)** Patient 30 (67 years, male) carried heterozygous c.5079G>A and c.7613C>T substitutions in the *NUP214* and *RYR3* genes, resulting in p. Gly1727Arg (NM_005085.4) and p. Thr2538Met (NM_001036.6); **(K)** Patient 29 (36 years, male) carried a heterozygous c.14585G>A substitution in exon 37 of the *RYR3* gene, resulting in p. Arg4862His (NM_001036.6); **(L)** Patient 14 (64 years, female) carried a heterozygous c.7789A>G substitution in exon 48 of the *RYR1* gene, resulting in p. Lys2597Glu (NM_000540.3); **(M)** Patient 13 (65 years, female) carried a heterozygous c.9559C>T substitution in exon 65 of the *RYR1* gene, resulting in p. Arg3187Trp (NM_000540.3); **(N)** Patient 24 (34 years, female) carried a heterozygous c.2020+1delG deletion in intron 19 of the *CAPN2* gene, resulting in p. X674_splice (NM_001748.5); **(O)** Patient 24 (29 years, female) carried a heterozygous c.111delC deletion in exon 2 of the *WNT16* gene, resulting in p. S38Pfs*15 (NM_057168.2).
