# Supplementary figures and images for: Clinical Characteristics and Pathogenic Gene Identification in Chinese Patients With Paget’s Disease of Bone
Source: Front Endocrinol (Lausanne). 2022 Mar 9;13:850462. doi: 10.3389/fendo.2022.850462 (PMC8959906; doi:10.3389/fendo.2022.850462)

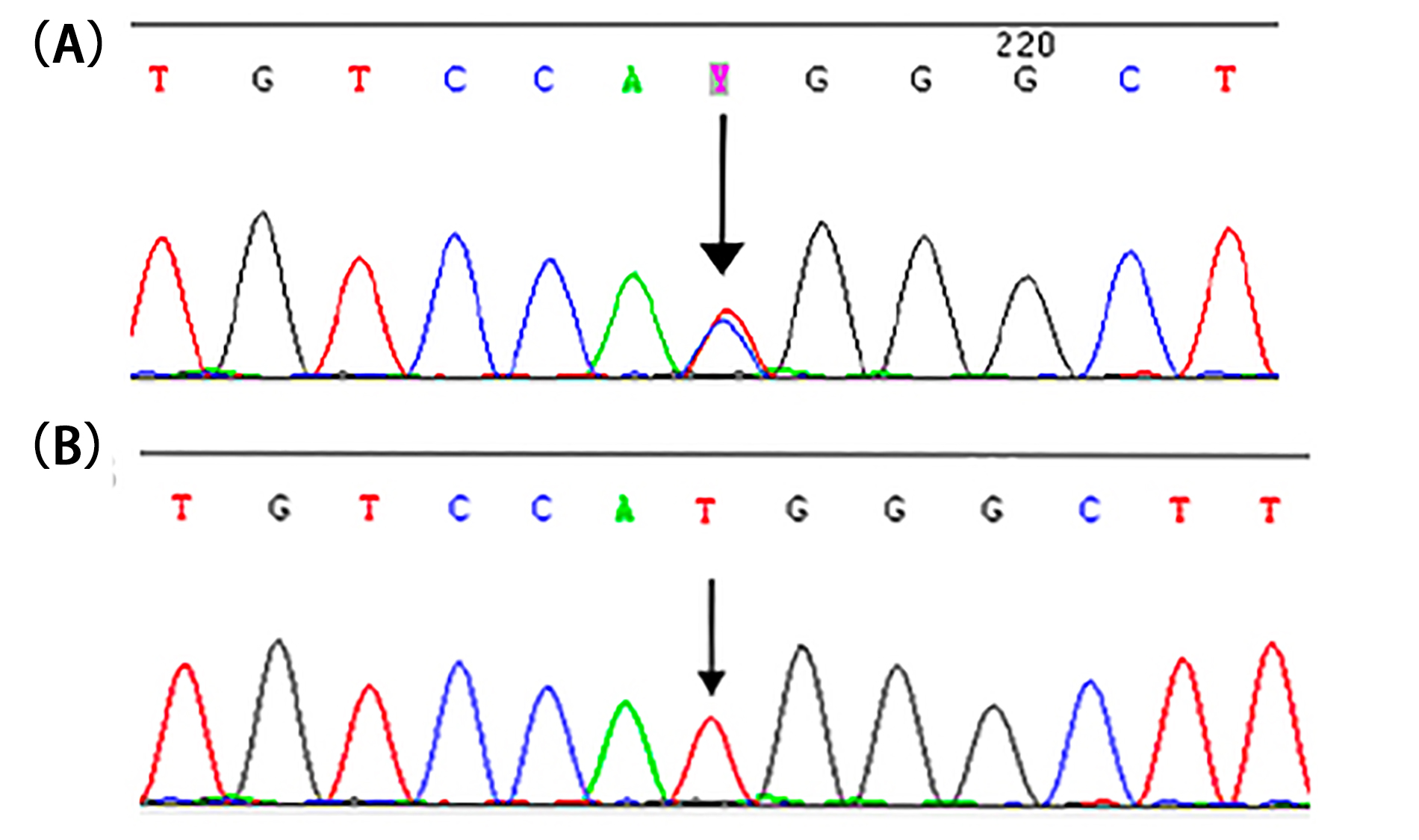

Supplement: Supplementary Figure 1 — Sequencing analysis of SQSTM1 in patients and healthy controls. (A) A 54-year-old woman harbored a heterozygous c.1211T>C transversion on exon 8 in SQSTM1, resulting in p. Met404Thr (NM_003900.5); (B) No M404T mutation was detected in healthy controls. [file Image_1.jpeg]

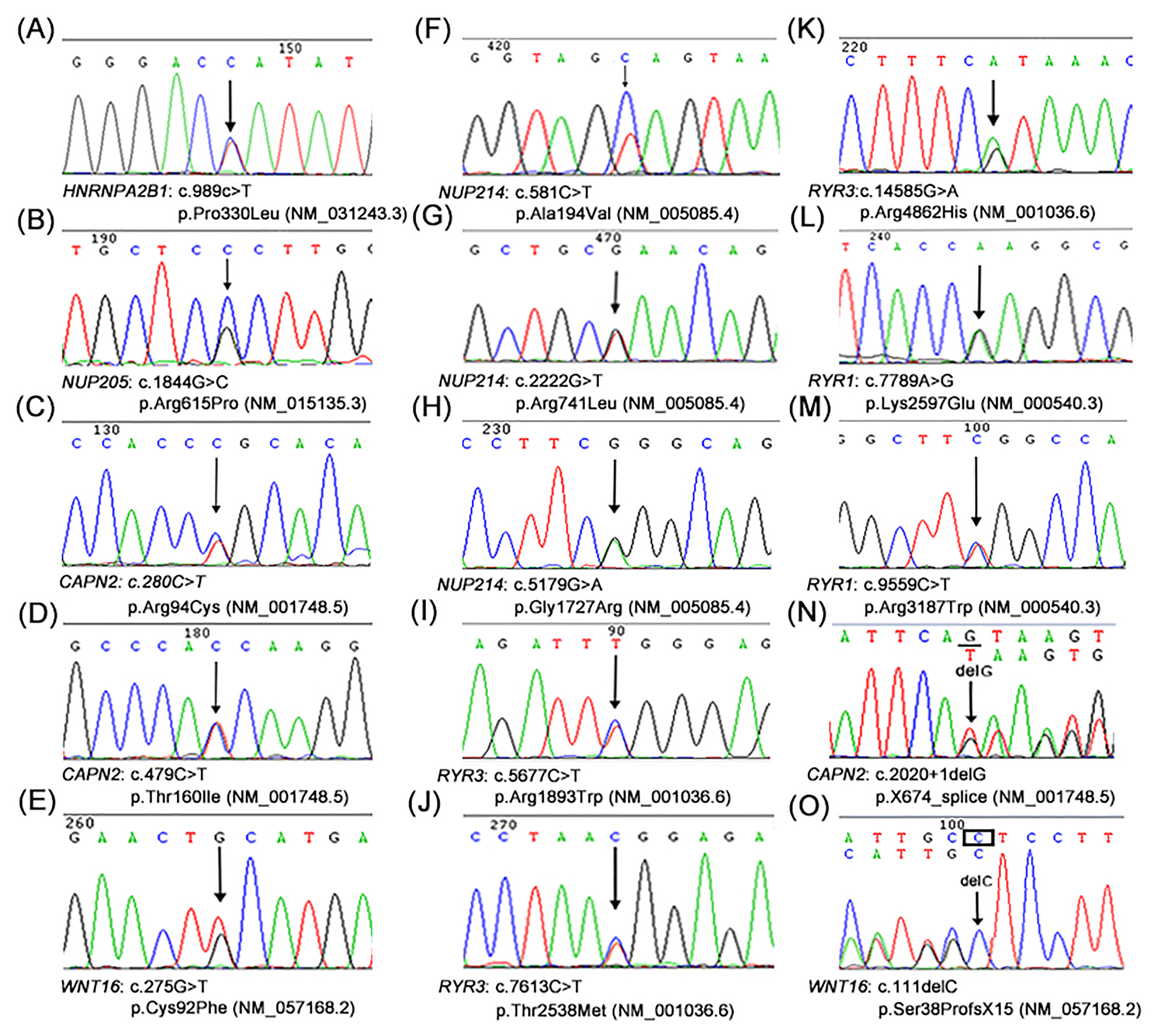

Supplement: Supplementary Figure 2 — Sequencing traces of the variants in the WNT16, RYR3, RYR1, HNRNPA2B1, NUP205, CAPN2 and NUP214 genes. (A) Patient 4 (32 years, male) carried a heterozygous c.989c>T substitution in exon 10 of the HNRNPA2B1 gene, resulting in p. Pro330Leu (NM_057168.2); (B) Patient 26 (80 years, female) carried a heterozygous c.1844G>C substitution in exon 13 of the NUP205 gene, resulting in p. Arg615Pro (NM_015035.3); (C) Patient 27 (82 years, male) carried a heterozygous c.280C>T substitution in exon 2 of the CAPN2 gene, resulting in p. (D, I) Patient 12 (83 years, female) carried heterozygous c.479C>T and c.5677C>T substitutions in the CAPN2 and RYR3 genes, resulting in p. Thr160Ile (NM_001748.5) and p. Arg1893Trp (NM_001036.6); (E) Patient 4 (42 years, female) carried a heterozygous c.275G>T substitution in exon 2 of the WNT16 gene, resulting in p. Cys92Phe (NM_057168.2); (F) Patient 28 (29 years, male) carried a heterozygous c.581C>T substitution in exon 4 of the NUP214 gene, resulting in p. A194Val (NM_005085.4); (G) Patient 30 (55 years, male) carried a heterozygous c.2222G>T substitution in exon 16 of the NUP214 gene, resulting in p. Arg741Leu (NM_005085.4); (H, J) Patient 30 (67 years, male) carried heterozygous c.5079G>A and c.7613C>T substitutions in the NUP214 and RYR3 genes, resulting in p. Gly1727Arg (NM_005085.4) and p. Thr2538Met (NM_001036.6); (K) Patient 29 (36 years, male) carried a heterozygous c.14585G>A substitution in exon 37 of the RYR3 gene, resulting in p. Arg4862His (NM_001036.6); (L) Patient 14 (64 years, female) carried a heterozygous c.7789A>G substitution in exon 48 of the RYR1 gene, resulting in p. Lys2597Glu (NM_000540.3); (M) Patient 13 (65 years, female) carried a heterozygous c.9559C>T substitution in exon 65 of the RYR1 gene, resulting in p. Arg3187Trp (NM_000540.3); (N) Patient 24 (34 years, female) carried a heterozygous c.2020+1delG deletion in intron 19 of the CAPN2 gene, resulting in p. X674_splice (NM_001748.5); (O) Patient 24 (29 years, fe [file Image_2.jpeg]
